# Impact of Late Ventricular Arrhythmias on Cardiac Mortality in Patients with Acute Myocardial Infarction

**DOI:** 10.1155/2019/5345178

**Published:** 2019-07-08

**Authors:** Takuma Takada, Koki Shishido, Takahiro Hayashi, Shohei Yokota, Hirokazu Miyashita, Hiroaki Yokoyama, Takashi Nishimoto, Tomoki Ochiai, Noriaki Moriyama, Kazuki Tobita, Shingo Mizuno, Futoshi Yamanaka, Masato Murakami, Yutaka Tanaka, Saeko Takahashi, Shigeru Saito

**Affiliations:** Department of Cardiology and Catheterization Laboratories, Shonan Kamakura General Hospital, Okamoto 1370-1, Kamakura City 247-8533, Japan

## Abstract

**Objectives:**

This study investigated the relationship between the timing of ventricular tachycardia or ventricular fibrillation (VT or VF) and prognosis in patients undergoing primary percutaneous coronary intervention (PCI) for acute myocardial infarction (AMI).

**Background:**

It is unknown whether the timing of VT/VF occurrence affects the prognosis of patients with AMI.

**Methods:**

From January 2004 to December 2014, 1004 patients with AMI underwent primary PCI. Of these patients, 888 did not have VT/VF (non-VT/VF group) and 116 had sustained VT/VF during prehospitalization or hospitalization. Patients with VT/VF were divided into two groups: early VT/VF (VT/VF occurrence before and within 2 days of admission, 92 patients) and late VT/VF (VT/VF occurrence >2 days after admission; 24 patients) groups.

**Results:**

The frequency of VT/VF occurrence was high between the day of admission and the 2nd day and between days 6 and 10 of hospitalization. The late VT/VF group had a significantly longer onset-to-balloon time, lower ejection fraction, poorer renal function, and higher creatine phosphokinase (CK)-MB level on admission (p< 0.001). They also had a lower 30-day cardiac survival rate than the early VT/VF and non-VT/VF groups (42% vs. 76% vs. 96%, p < 0.001). Moreover, independent predictors of in-hospital cardiac mortality among patients with AMI who had sustained VT/VF were higher peak CK-MB [Odds ratio (OR: 1.001, 95%confidence interval (CI): 1.000-1.002, p= 0.03)], higher Killip class (OR: 1.484, 95%CI 1.017-2.165, p= 0.04), and late VT/VF (OR: 3.436, 95%CI 1.115-10.59, p= 0.03).

**Conclusions:**

The timing of VT/VF occurrences had a bimodal peak. Although late VT/VF occurrence after primary PCI was less frequent than early VT/VF occurrence, patients with late VT/VF had a very poor prognosis.

## 1. Introduction

Ventricular tachycardia (VT) and ventricular fibrillation (VF) are fatal arrhythmias that could occur in cardiac collapse and are major complications of acute myocardial infarction (AMI) [1, 2]. The majority of VT/VF occurrences in patients with AMI are within 48 h after AMI symptom onset [3]. Several previous studies have suggested that VT/VF occurrence is associated with poor in-hospital and long-term outcomes, irrespective of the timing of occurrence, i.e., within 48 h or >48 h after symptom onset [4–7]. However, the analyses in these studies were performed before the era of primary percutaneous coronary intervention (PCI). Therefore, data regarding the relationship between the timing of VT/VF and prognosis in patients undergoing primary PCI for AMI are limited.

The purpose of this study was to investigate the timing of VT/VF occurrences and to compare in-hospital cardiac mortality between patients with VT/VF, which occurred before and within 2 days (48 h) after admission (early VT/VF), and patients with VT/VF, which occurred >2 days after admission (late VT/VF).

## 2. Methods

### 2.1. Study Population

From January 2004 to December 2014, 1121 consecutive patients with AMI presented to our institution. Patients were included in these investigations if they were 18 years or older, presented with AMI within 24 hours of their symptom onset, and underwent primary PCI. Definitions of AMI and the standard of care were established according to the latest guidelines at that time. The AMI guideline had been modified in 2008 and 2013 in Japan. It showed there are minor changes; diagnosis of AMI is almost unchanged. The diagnosis of AMI definitions showed as typical lasting chest pain, increase of cardiac enzymes above the normal range, and onset of ST-T changes compatible with myocardial ischemia (ST segment elevation or depression, T-wave inversion) or abnormal Q waves. Emergent coronary angiography (CAG) and primary PCI were performed using standard techniques. Patients were excluded from this study if they received continuous hemodialysis, had asystole or pulseless electrical activity in the emergency room and, thus, were not taken to the catheter laboratory, underwent emergent coronary artery bypass graft surgery, had ventricular septal perforation, had cardiac rupture and required emergent surgery, or had incomplete data. Our study was a retrospective research. Written informed consent to perform the procedure as well as using the data retrospectively as registry was obtained from all patients. This study design was approved by the institutional review board at Tokushukai Group Ethical Committee (Approval Number: TGE00989-024), and it conformed to the Declaration of Helsinki.

### 2.2. Definitions of VT/VF and Classification of the Patients

We investigated patients who had VT/VF during emergent transportation, in the emergency room or catheter laboratory room, or during hospitalization after primary PCI. Patients with sustained VT/VF received cardiopulmonary resuscitation. Sustained VT was defined as a regular wide-complex tachycardia of ventricular origin, lasting ≥30 seconds or accompanied by hemodynamic compromise requiring electrical cardioversion. VF was defined as irregular waves of inconsistent shape, without distinct QRS complexes, or T-waves. VT or VF was diagnosed using a twelve-lead electrocardiogram or based on electrocardiogram monitoring during chart review. We included patients with sustained VT/VF event before PCI, during PCI and after PCI in VT/VF group. Patients who had only nonsustained VT with stable hemodynamics were not classified as patients with VT/VF; they were included in the non-VT/VF group. We defined cardiac mortality as death due to myocardial ischemia and infarction, cardiac tamponade and worsening heart failure, procedure-related death, valve-related death, sudden or unwitnessed death and death of unknown cause.

Initially, the patients were divided into those with VT/VF and those without VT/VF. Subsequently, patients with VT/VF were further divided into two groups according to the timing of the arrhythmias: patients with VT/VF occurring within 2 days of hospital admission (early VT/VF) and those with VT/VF occurring >2 days after hospital admission (late VT/VF). Patients with episodes of early and late VT/VF were included in late VT/VF group.

### 2.3. Clinical Outcomes

Thirty-day cardiac mortality was compared between patients with VT/VF and those without VT/VF. Thereafter, it was compared between the early and late VT/VF groups. Moreover, we also investigated the timing of VT/VF occurrences and in-hospital cardiac death. Finally, we researched the independent predictors for the in-hospital cardiac mortality in patients with VT/VF.

### 2.4. Statistical Analysis

We compared continuous variables using Student's t-test or Mann–Whitney U test. Fisher's exact test was used for categorical variables. P value < 0.05 was considered statistically significant. Baseline variables in the univariate analysis, with p value < 0.05, were considered in the multivariate analysis, which was performed to determine independent predictors of late VT/VF using a logistic-regression model. In addition, multivariate Cox hazard regression model was constructed to investigate the predictor for in-hospital death. The variables examined in these analyses were over age 75 year of age, onset-to-balloon time, Killip class, creatine phosphokinase (CK)-MB on admission, estimated glomerular filtration rate (eGFR), ejection fraction (EF), peak CK-MB and late VT/VF. In this study, the start of the follow-up was the timing of primary PCI. We defined that long follow-up periods were one year from primary PCI. The long follow-up outcomes adjusted the duration on risk of events with log-rank analysis. Survival curves regarding 30-day cardiac mortality were estimated using the Kaplan-Meier method and survival estimates were also compared using the log-rank test. All statistical analyses were performed using EZR (Saitama Medical Center, Jichi Medical University) [8], which is a graphical user interface for R (the R Foundation for Statistical Computing, Vienna, Austria).

## 3. Results

### 3.1. Study Flow Chart

**Figure 1:**
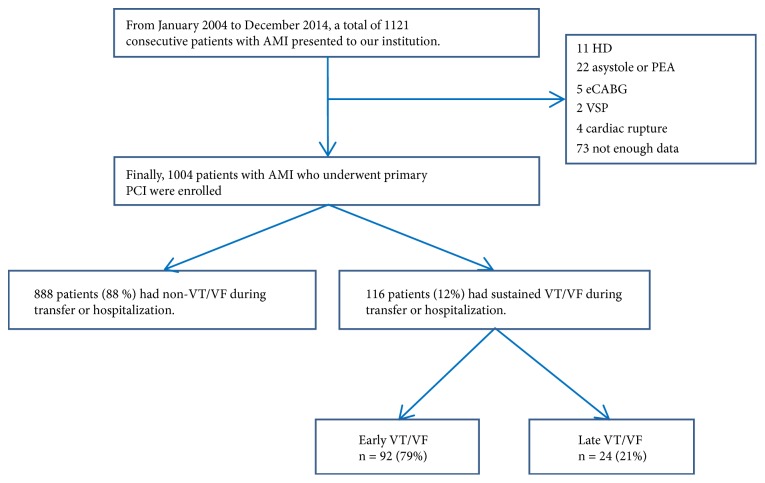
Study flow chart. eCABG: emergent coronary bypass graft surgery, HD: hemodialysis, PCI: percutaneous coronary intervention, PEA: pulseless electrical activity, VSP: ventricular septal perforation, VT/VF: ventricular tachycardia or ventricular fibrillation.

From January 2004 to December 2014, 1004 patients with AMI who underwent primary PCI were enrolled. Of these patients, 888 (88%) did not have VT/VF while 116 (12%) had VT/VF during prehospitalization or hospitalization (Figure 1). Eleven patients who received continuous hemodialysis and 22 patients who had asystole or pulseless electrical activity in the emergency room and, thus, were not taken to the catheter laboratory were excluded from this study. We also excluded five patients who underwent emergent coronary artery bypass graft surgery, two patients who had ventricular septal perforation, four patients who had cardiac rupture and required emergent surgery, and 73 patients who had incomplete data.

### 3.2. Comparison of the Patients with VT/VF and Those without VT/VF

Baseline characteristics of patients with VT/VF and those of patients without VT/VF are shown in Table 1. Patients with VT/VF had a lower systolic blood pressure, higher heart rate, higher Killip class, lower eGFR, and a lower EF on admission.

### 3.3. Comparison of the Patients with Early VT/VF and Late VT/VF

According to the timing of VT/VF occurrence, 92 patients had early VT/VF (79%) and 24 patients had late VT/VF (21%). Their baseline characteristics are shown in Table 1. Patients in the early VT/VF group were younger than those in the late VT/VF group (69 ± 12 vs. 77 ± 11 years, p= 0.002). No significant differences in clinical history were noted. Onset-to-balloon time in the early VT/VF group was shorter than that in the late VT/VF group (3.6 ± 2.6 vs. 7.8 ± 6.5 h, p< 0.001). Other factors such as systolic blood pressure in the emergency room (early VT/VF vs. late VT/VF: 121 ± 37 vs. 111 ± 29 mmHg, p= 0.27), the ratio of bystander cardiopulmonary resuscitation (93% vs. 100%, p= 0.34), the ratio of ST elevated myocardial infarction (92% vs. 83%, p=0.24), and Killip class (p= 0.63) did not differ between the groups. Compared with patients with early VT/VF, patients with late VT/VF had a higher heart rate, higher CK-MB level, poorer renal function, and lower ejection fraction on admission. Troponin I level on admission was statistically lower in early VT/VF group than that in late VT/VF group (1.8 ± 6.3 vs. 11.2 ± 16.6 ng/mL, p< 0.001). In both groups, no patient had received a *β*-blocker before AMI onset.

Angiographic findings in patients with early and late VT/VF are shown in Table 2. No significant differences in the culprit vessels, preprocedural Thrombolysis in Myocardial Infarction (TIMI) flow, and after procedural TIMI flow were observed between patients with early VT/VF and those with late VT/VF.

The clinical outcomes of patients with early and late VT/VF are shown in Table 3. The peak CK and peak CK-MB levels were comparable between the two groups. Length of hospital stay tended to be longer in the late VT/VF group; however, no statistically significant differences were noted between the two groups. Moreover, the late VT/VF group had a significantly higher in-hospital cardiac mortality rate than the early VT/VF group (58 vs. 24%, p= 0.002). The occurrence of recurrent myocardial infarction and stroke did not differ between the two groups during hospitalization.

The factor associated with an increased risk of late VT/VF was onset-to-balloon time after multivariable adjustment with a significant level of p< 0.05 in Table 1 (OR 2.03, 95% CI 1.01–4.08, p= 0.047) (Table 4). Further, of the 116 patients with VT/VF, 36 patients (31%) died of a cardiac-related cause during hospitalization. Multivariate Cox regression model for in-hospital cardiac death (Table 5) revealed that late VT/VF was one of independent predictors (OR 3.436, 95% CI 1.115–10.59, p= 0.03).

### 3.4. The 30-Day Cardiac Survival Rates

**Figure 2:**
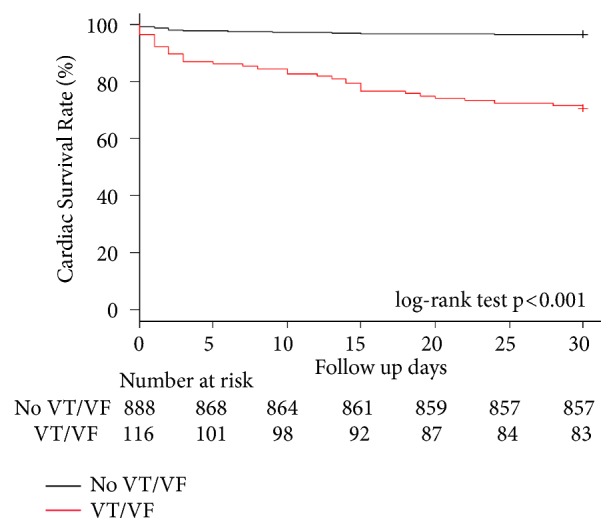
Clinical outcomes regarding cardiac survival rate between patients without VT/VF and those with VT/VF during the 30-day period. Notes: log-rank test p <0.001 for comparisons between patients without VT/VF and those with VT/VF. VT/VF: ventricular tachycardia or ventricular fibrillation.

**Figure 3:**
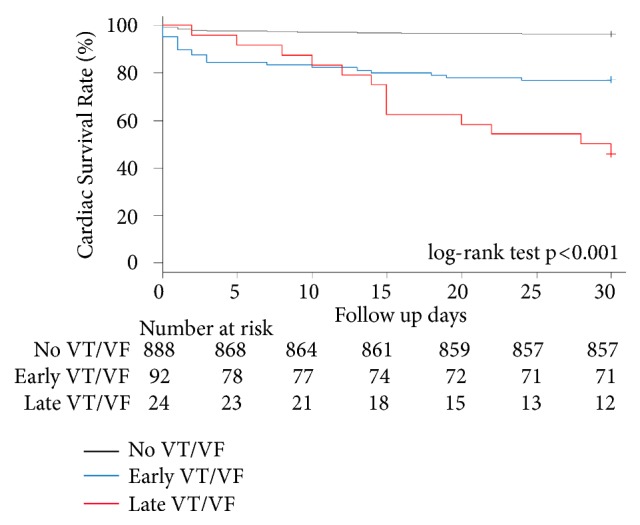
Clinical outcomes regarding cardiac survival rate among patients without VT/VF, those with early VT/VF, and those with late VT/VF during the 30-day period. Notes: log-rank test p <0.001 for comparisons between patients without VT/VF and those with early VT/VF, those without VT/VF, and those with late VT/VF, and between those with early VT/VF and those with late VT/VF. VT/VF: ventricular tachycardia or ventricular fibrillation.

The Kaplan-Meier curve demonstrated the cardiac survival rate in the patients with VT/VF compared with those patients without VT/VF during a 30-days (Figure 2). The 30-day cardiac survival rate in patients with VT/VF was significantly lower than those patients without VT/VF (69 vs. 96%, p< 0.001). The 30-day cardiac survival rates in patients without VT/VF, patients with early VT/VF, and patients with late VT/VF are shown in Figure 3. The Kaplan-Meier curve demonstrated statistically significant differences in (30-day) survival rate among the three groups. The (30-day) survival rate of patients without VT/VF was 96%, that of patients with early VT/VF was 76%, and that of patients with late VT/VF was 42% (p< 0.001).

### 3.5. Timing and Frequency of Late VT/VF

**Figure 4:**
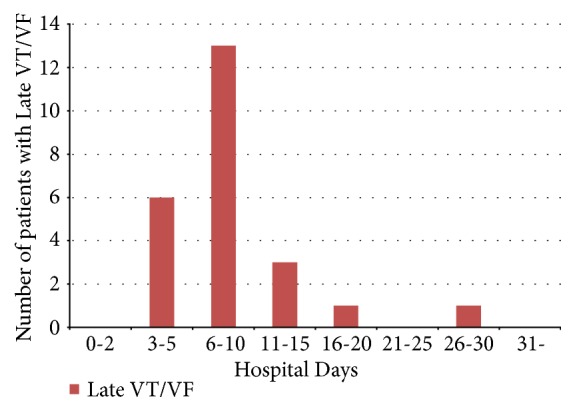
Timing and frequency of late VT/VF. VT/VF: ventricular tachycardia or ventricular fibrillation.

In early VT/VF group (92 patients), fifty-one patients had the occurrences of VT/VF before PCI, 33 patients had the occurrences of VT/VF during PCI, and 8 patients had the occurrences of VT/VF after PCI within 48 hours after admission. Eighty-nine patients with VT/VF (77%) had the timing of VT/VF occurrences within 24 h of admission. Of these, 13 patients had the occurrence of VT/VF within 1 hour from onset. The frequency of late VT/VF was high between the 6th and 10th days of hospitalization (Figure 4). In late VT/VF group (24 patients), re-CAG was performed for 9 patients (38%). Of these, 8 patients had no stent thrombosis, no reinfarction and no other significant stenosis. Only 1 patient had subacute stent thrombosis and underwent emergent re-PCI. In the other 15 patients (62%) of late VT/VF group, re-CAG was not performed. In 4 patients of the 15 patients, VT/VF was stopped immediately by the appropriate advanced cardiovascular life support. ECG and other noninvasive test after the resuscitation showed no ischemic change. Two patients (8%) had cardiac tamponade and died. The rest 9 patients (38%) had pulseless electrical activity during the cardiopulmonary resuscitation. Their family did not hope the additional treatments included reangiography or percutaneous cardiopulmonary support, and these patients died.

### 3.6. Timing and Frequency of In-Hospital Cardiac Death

**Figure 5:**
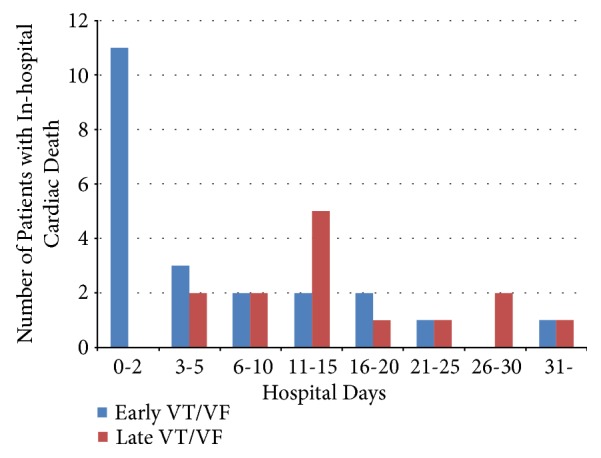
Timing and frequency of in-hospital cardiac death. VT/VF: ventricular tachycardia or ventricular fibrillation.

The relationship between early or late VT/VF and in-hospital cardiac death was demonstrated in Figure 5. Overall, the histogram regarding in-hospital cardiac death showed a bimodal peak, within 2 days of admission and between days 11 and 15 of admission.

## 4. Discussion

This study compared the timing of VT/VF occurrences in patients with AMI who underwent primary PCI and 30-day cardiac mortality between patients without VT/VF, those with early VT/VF, and those with late VT/VF. The main findings of this study are as follows: (1) the timing of VT/VF occurrences had two peaks, i.e., within 2nd day and around 8 days after admission and (2) patients with late VT/VF had a very poor prognosis compared to that of patients without VT/VF and patients with early VT/VF.

Of 1004 patients with AMI who were hospitalized within 24 h of AMI symptom onset and underwent primary PCI, 12% had VT/VF in this study. Previous AMI registry data, including the APEX-AMI and HORIZONS-AMI trials, demonstrated that VT/VF incidence in the acute phase of AMI was higher in our study (APEX-AMI 5.7% and HORIZONS-AMI 5.2%) [3, 9]. Our study included patients with VT/VF, which occurred during emergency transportation, and our study population included a higher number of patients with Killip class III/IV (48%) than that of previous studies. Moreover, a majority of the VT/VF incidences occurred within 48 h after admission in our study, which was in line with a previous study on AMI [1, 2, 9].

Furthermore, our study showed that the 30-day mortality rate of patients without VT/VF vs. patients with early VT/VF vs. patients with late VT/VF was 3.9% vs. 24% vs. 58% and that VT/VF was significantly associated with high 30-day mortality. These findings are acceptable in daily clinical practice and were reported in various studies [10, 11]. Patients with late VT/VF had worse outcomes during hospitalization; this finding is consistent with that of a previous study [3, 12]. Podolecki et al. [12] reported the similar results of worse outcome in patients with ischemic or infarction related ventricular arrhythmia (IVA) starting after 48 hours from recanalization. Comparing the differences between the results of the prior and our study, they did not research a cause of death such as cardiac mortality or noncardiac mortality for patients with late VT/VF. We investigated all patients regarding in-hospital recurrent MI, stroke, cardiac mortality, and noncardiac mortality as shown in Table 3. Although assessing detailed patient information from previous large registry studies was difficult, we reviewed the charts of each patient in detail. Our study demonstrated that the frequency of late VT/VF was high on days 6 to 10 after hospitalization and that the timing of in-hospital cardiac death had a bimodal peak. To the best of our knowledge, this is the first study to demonstrate that VT/VF occurrence has a bimodal peak after primary PCI in patients with AMI. Moreover, the timing of late VT/VF was strongly related to the timing of the secondary peak of in-hospital cardiac death, and late VT/VF was an independent predictor of in-hospital cardiac mortality in the multivariate analysis.

According to a previous report, early VF is triggered by acute ischemia in combination with an elevated sympathetic tone due to total coronary artery occlusion [13–15]. Sudden cardiac death has also been associated with a higher sympathetic tone [16, 17]. After reperfusion, ventricular arrhythmia due to toxic metabolites and ions, such as lactate and potassium, which flow into the peripheral coronary artery or due to mitochondrial dysfunction induced by myocardial ischemia, occurs [18]. Late VF could result from severe heart failure; however, the mechanism and reason for late VF remain unknown [17, 19]. In our study, patients with early VT/VF had a shorter onset-to-balloon time than those with late VT/VF, which possibly indicates that the time after coronary artery occlusion was shorter and that the main pathophysiology of early VT/VF could be related to an elevated sympathetic tone. In contrast, patients with late VT/VF had a longer onset-to-balloon time and higher brain natriuretic peptide levels on admission, which could mean that the main pathophysiology of VT/VF was altered with the passage of ischemic time and was affected by heart failure. Although the trigger is not completely clear, some patients suddenly experienced late VT/VF related by R-on-T phenomenon. The patients with late VT/VF had various worse conditions. For example, some patients had decompensated heart failure, some patients had cardiac tamponade, and the other patients had electrolyte abnormality. A few patients had hypomagnesemia when late VT/VF occurred. We considered that the main pathophysiology of VT/VF was the effect of myocardial injury due to ischemia for the first 5 days. Subsequently, late VT/VF was caused by pressure- or volume-overload due to severe heart failure or the arrhythmogenic substrate which was attributed to various electrophysiological or structural changes in the scar tissue of the ventricular muscle. In previous animal experiments regarding the mechanism of IVA [20], IVA occurred in two distinct phases. Phase 1 was from a few minutes to 0.5 or 1 hour after onset of AMI and the reversible phase of ischemic injury, which might be applicable to patients having the occurrences of VT/VF before PCI. Whereas phase 2 IVAs were the infarct evolution phase, to be associated with the reperfusion of ischemic areas and beginning at approximately 1.5 to 5 hours after the onset of AMI and lasting 2 to 3 days. Moreover, they divided phase 2 into two sources: abnormal automaticity and reentry. We considered that the occurrences of VT/VF during and after PCI within 48 hours might be included in phase 2 due to abnormal automaticity because early VT/VF was triggered by acute ischemia with the elevated sympathetic tone. On the other hand, late VT/VF might be included in phase 2 due to reentry because we considered that late VT/VF was caused by various electrophysiological or structural changes with myocardial necrosis. We also suggested that there were differences of phase 2 between pig and human because patients were influenced by reperfusion therapy with PCI.

Hence, the pathophysiology of VT/VF in the two groups was different. Even if reperfusion therapy was successful, in cases of long onset-to-balloon time, careful management using electrocardiogram monitoring is necessary, considering the low survival rate of patients with late VT/VF. Although *β*-blocker, amiodarone, and wearable ICD might improve their clinical outcomes, we could not investigate the effect of these managements for patients with late VT/VF. Referring AHA/ACC/HRS guideline 2017, wearable ICD was recommended for the patients who were within 40 days from an MI or revascularization within the past 90 days (class IIb) [21]. It may prevent patients from the risk of sudden cardiac death in the early phase after revascularization to allow time for recovery of ventricular function [22]. We could not simply compare the previous study to ours because the baseline characteristics of the previous study were not similar to our study. However, unlike patients with early VT/VF, patients with late VT/VF were in a worse condition, including older age, poorer renal function, lower hemoglobin levels, and left ventricular ejection fraction. It might affect that they could not be discharged from the hospital within 1 week. We consider that such patients with late VT/VF may be recommended to use wearable ICD for the clinical implications because late VT/VF had a poor prognosis. Further investigation is warranted for the better management of patients with late VT/VF.

## 5. Limitations

Our study was a retrospective, nonrandomized, single-center research. In addition, the number of patients who had late VT/VF was relatively small. We could not provide more detail on the types of VT/VF, such as fast, slow, monomorphic, or polymorphic. Our study could not distinguish between VT and VF regarding prognosis. It was difficult to distinguish between the patients with VT and those with VF completely because these types of arrhythmia were overlapping in almost all cases. Previous large scale studies such as HORIZONS-AMI Trial [9] and TIMI Phase II trial [10] also did not distinguish between VT and VF regarding prognosis. We followed the methods of these previous researches for our study. For the long study period, including minor changes of devices for PCI was considered. In addition, we did not include patients who died by VT/VF with AMI before PCI. This would influence the results and interpretation of our study, but it was impossible to count the patients because they died outside of hospital. Furthermore, the multivariate analysis shown in Table 4 might be overfitting because eight factors were fitted for the adjustment. Larger prospective trials are needed to confirm our findings.

## 6. Conclusions

In this study, the timing of VT/VF had two peaks, and late VT/VF was strongly related to the timing of the secondary peak of in-hospital cardiac death. Careful management of patients with late VT/VF is crucial because they have a very poor prognosis.

## Figures and Tables

**Table 1:** Baseline characteristics of the patients with VT/VF or no VT/VF and early VT/VF or late VT/VF.

Characteristics	VT/VF (n=116)	No VT/VF (n=888)	p value	Early VT/VF (n=92)	Late VT/VF (n=24)	p value
Age, y	70 ± 12	70 ± 13	0.70	69 ± 12	77 ± 11	0.002
Male, n (%)	85 (73)	639 (72)	0.74	71 (77)	14 (58)	0.074
BMI	22 ± 3.9	23 ± 4.0	0.81	23 ± 4.0	24 ± 3.5	0.38
*Clinical history*						
Hypertension, n (%)	63 (54)	515 (58)	0.37	48 (52)	14 (61)	0.49
Diabetes mellitus, n (%)	41 (35)	284 (32)	0.46	32 (35)	8 (32)	0.46
Dyslipidemia, n (%)	50 (43)	442 (52)	0.20	30 (33)	11 (48)	0.48
Smoking, n (%)	48 (41)	400 (45)	0.48	38 (42)	9 (39)	1
FH of CAD, n (%)	15 (13)	142 (16)	0.41	10 (11)	4 (17)	0.48
prior MI, n (%)	13 (11)	80 (9)	0.50	10 (11)	3 (13)	0.73
prior PCI, n (%)	20 (17)	115 (13)	0.19	16 (17)	4 (17)	1.00
*Presenting data*						
SBP, mmHg	118 ± 35	135 ± 32	< 0.001	121 ± 37	111 ± 29	0.27
HR, beats/min	82 ± 29	75 ± 21	0.004	78 ± 27	97 ± 30	0.004
Onset to balloon time, h	4.5 ± 4.1	5.0 ± 4.2	0.18	3.6 ± 2.6	7.8 ± 6.5	< 0.001
Killip class			< 0.001			0.63
I, n (%)	40 (34)	665 (75)		32 (35)	8 (33)	
II, n (%)	20 (17)	106 (12)		15 (16)	5 (21)	
III, n (%)	13 (11)	55 (6.3)		9 (9.8)	4 (17)	
IV, n (%)	43 (37)	51 (5.8)		36 (39)	7 (29)	
CK, U/L	506 ± 1052	430 ± 922	0.41	303 ± 478	1283 ± 1958	< 0.001
CK-MB, U/L	50 ± 73	43 ± 62	0.28	38 ± 57	95 ± 105	< 0.001
Hb, g/dL	13 ± 2.3	14 ± 2.1	0.015	14 ± 1.9	12 ± 3.3	0.007
eGFR, ml/min/1.73 m^2^	54 ± 18	62 ± 20	< 0.001	58 ± 17	40 ± 16	< 0.001
BNP, pg/dL	328 ± 550	172 ± 388	0.002	250 ± 528	619 ± 553	0.016
EF, %	47 ± 14	55 ± 12	< 0.001	49 ± 13	41 ± 17	0.019

BMI: body mass index, BNP: brain natriuretic peptide, CAD: coronary artery disease, CK: creatine kinase, CK-MB: creatine kinase-MB, EF: ejection fraction, eGFR: estimated glomerular filtration rate, FH: family history, Hb: hemoglobin, HR: heart rate, MI: myocardial infarction, PCI: percutaneous coronary intervention, SBP: systolic blood pressure, VT/VF: ventricular tachycardia or ventricular fibrillation.

**Table 2:** Angiographic findings of the patients with early VT/VF or late VT/VF.

Characteristics	Early VT/VF (n=92)	Late VT/VF (n=24)	p value
LMT, n (%)	7 (7.6)	1 (4.2)	0.12
LAD, n (%)	40 (43)	8 (33)	
LCX, n (%)	4 (4.3)	5 (21)	
RCA, n (%)	41 (45)	10 (42)	
Multivessel disease, n (%)	36 (39)	15 (63)	0.063
CTO, n (%)	5 (5.4)	2 (8.3)	0.63
*Preprocedural TIMI flow*			
Grade 0, n (%)	74 (80)	19 (79)	1
Grade I, n (%)	4 (4.3)	1 (4.2)	
Grade II, n (%)	7 (7.6)	2 (8.3)	
Grade III, n (%)	7 (7.6)	2 (8.3)	
*Postprocedural TIMI flow*			
Grade 0, n (%)	1 (1.1)	0	0.54
Grade I, n (%)	2 (2.2)	0	
Grade II, n (%)	7 (7.6)	4 (17)	
Grade III, n (%)	82 (89)	20 (83)	
Aspiration, n (%)	78 (72)	20 (83)	0.78
IABP, n (%)	49 (53)	13 (54)	0.82
PCPS, n (%)	16 (17)	3 (13)	0.76

CTO: chronic total occlusion, IABP: Intra-aortic balloon pumping, LAD: left anterior descending coronary artery, LCX: left circumflex coronary artery, LMT: left main trunk, PCPS: percutaneous cardiopulmonary support, RCA: right coronary artery, TIMI: thrombolysis in myocardial infarction, VT/VF: ventricular tachycardia or ventricular fibrillation.

**Table 3:** Clinical outcomes of the patients with early VT/VF or late VT/VF.

Characteristics	Early VT/VF (n=92)	Late VT/VF (n=24)	p value
Peak CK U/L	4362 ± 4060	4817 ± 4170	0.63
Peak CK-MB U/L	321 ± 308	433 ± 544	0.20
Length of hospital stay, days	16 ± 29	25 ± 15	0.15
In-hospital recurrent MI, n (%)	2 (2.2)	1 (4.2)	0.51
In-hospital stroke, n (%)	1 (1.1)	1 (4.2)	0.37
In-hospital cardiac mortality, n (%)	22 (24)	14 (58)	0.002
In-hospital noncardiac mortality, n (%)	3 (3.2)	2 (8.3)	0.28
Recurrent MI at 1 year, n (%)	2 (2.2)	1 (4.2)	0.63
Stroke at 1 year, n (%)	3 (3.3)	0 (0)	0.58
All-cause mortality at 1 year, n (%)	26 (28)	17 (71)	< 0.001
Cardiac mortality at 1 year, n (%)	22 (24)	14 (58)	< 0.001

CK: creatine kinase, CK-MB: creatine kinase-MB, MI: myocardial infarction, VT/VF: ventricular tachycardia or ventricular fibrillation.

Note: P value for recurrent MI, stroke, all-cause mortality, and cardiac death were analyzed by log-rank test.

**Table 4:** Multivariable adjustment of risk factors of late VT/VF.

	OR	95% CI Lower	95% CI Upper	p value
Age	1.12	0.906	1.380	0.30
HR	1.02	0.983	1.050	0.35
Onset to balloon time	2.03	1.01	4.080	0.047
CK-MB	1.01	0.984	1.030	0.64
Hb	0.423	0.216	0.827	0.012
eGFR	0.847	0.741	0.968	0.015
BNP	0.998	0.995	1.000	0.064
EF	0.994	0.916	1.080	0.88

BNP: brain natriuretic peptide, CI: confidence interval, CK-MB: creatine kinase-MB, EF: ejection fraction, eGFR: estimated glomerular filtration rate, HR: heart rate, OR: odds ratio, VT/VF: ventricular tachycardia or ventricular fibrillation.

**Table 5:** The independent predictors for in-hospital cardiac death-Cox regression model.

	OR	95% CI Lower	95% CI Upper	p value
Age >=75 years	1.397	0.532	3.667	0.50
Onset to balloon time	0.987	0.898	1.084	0.78
Killip class	1.484	1.017	2.165	0.04
EF	0.986	0.959	1.015	0.34
eGFR	0.986	0.950	1.024	0.46
Peak CK-MB	1.001	1.000	1.002	0.03
Late VT/VF	3.436	1.115	10.59	0.03

CI: confidence interval, CK-MB: creatine kinase-MB, EF: ejection fraction, eGFR: estimated glomerular filtration rate, OR: odds ratio, VT/VF: ventricular tachycardia or ventricular fibrillation.

## Data Availability

(1) The retrospective research data used to support the findings of this study have been deposited in the Department of Cardiology and Catheterization Laboratories, Shonan Kamakura General Hospital Repository.
